# Causal associations between modifiable risk factors and isolated REM sleep behavior disorder: a mendelian randomization study

**DOI:** 10.3389/fneur.2024.1321216

**Published:** 2024-02-07

**Authors:** Ru-Yu Zhang, Fu-Jia Li, Qian Zhang, Li-Hong Xin, Jing-Ying Huang, Jie Zhao

**Affiliations:** ^1^Department of Respiratory and Critical Care Medicine, The Second Affiliated Hospital of Xuzhou Medical University, Xuzhou, China; ^2^Department of Neurology, The Affiliated Hospital of Xuzhou Medical University, Xuzhou, China

**Keywords:** mendelian randomization, isolated REM sleep behavior disorder, risk factors, causal association, genetic correlation

## Abstract

**Objectives:**

This Mendelian randomization (MR) study identified modifiable risk factors for isolated rapid eye movement sleep behavior disorder (iRBD).

**Methods:**

Genome-wide association study (GWAS) datasets for 29 modifiable risk factors for iRBD in discovery and replication stages were used. GWAS data for iRBD cases were obtained from the International RBD Study Group. The inverse variance weighted (IVW) method was primarily employed to explore causality, with supplementary analyses used to verify the robustness of IVW findings. Co-localization analysis further substantiated causal associations identified via MR. Genetic correlations between mental illness and iRBD were identified using trait covariance, linkage disequilibrium score regression, and co-localization analyses.

**Results:**

Our study revealed causal associations between sun exposure-related factors and iRBD. Utilizing sun protection (odds ratio [OR] = 0.31 [0.14, 0.69], *p* = 0.004), ease of sunburn (OR = 0.70 [0.57, 0.87], *p* = 0.001), childhood sunburn occasions (OR = 0.58 [0.39, 0.87], *p* = 0.008), and phototoxic dermatitis (OR = 0.78 [0.66, 0.92], *p* = 0.003) decreased iRBD risk. Conversely, a deep skin color increased risk (OR = 1.42 [1.04, 1.93], *p* = 0.026). Smoking, alcohol consumption, low education levels, and mental illness were not risk factors for iRBD. Anxiety disorders and iRBD were genetically correlated.

**Conclusion:**

Our study does not corroborate previous findings that identified smoking, alcohol use, low education, and mental illness as risk factors for iRBD. Moreover, we found that excessive sun exposure elevates iRBD risk. These findings offer new insights for screening high-risk populations and devising preventive measures.

## 1 Introduction

Isolated rapid eye movement behavior disorder (iRBD) is defined as parasomnia characterized by the absence of muscle atonia during rapid eye movement (REM) sleep, often accompanied by dream enactment behavior (1, 2). Patients frequently display aggressive behaviors such as shouting, punching, or striking during sleep, leading to sleep disturbances and potential harm to themselves or their bed partners (3–5). Furthermore, previous studies have shown that iRBD is most importantly a potential preclinical sign of neurodegenerative synucleinopathies, with more than 80% of patients with iRBD eventually develop Parkinson’s disease (PD), dementia with Lewy bodies (DLB), or multiple system atrophy (MSA), (6–9). Therefore, identifying risk factors, especially those amenable to intervention, is crucial for screening high-risk populations and reducing the incidence of iRBD.

Numerous studies have suggested that increased sun exposure can reduce the incidence of PD (10–12), but its effect on iRBD is still uncertain. Previous observational studies have identified various risk factors for iRBD including lifestyle factors (smoking, alcohol consumption, coffee and tea intake, and low physical activity), low education levels, agricultural work, pesticide exposure, head injuries, mental illness, and antidepressant use (13–20). However, substantial contradictions and debates persist regarding these factors. In a multicenter case–control study, Postuma et al. found that patients with iRBD (diagnosed using polysomnography [PSG]) were more likely to report smoking, low educational levels, head injuries history, occupational pesticide exposure, and farming work (16). Additionally, a Canadian Longitudinal Study on Aging (CLSA) with a sample size of 30,097 individuals found mental illness and antidepressant use could also serve as risk factors for possible RBD (pRBD, diagnosed via an RBD single-questionnaire) (19). However, a study from Beijing conducted by Zhang et al. involving 7,225 individuals found no association between education levels, occupation, antidepressant treatment, and the incidence of pRBD (diagnosed using the RBD Questionnaire-Hong Kong) (20). Furthermore, a community-based study led by Jian-Fang Ma involving 3,635 individuals failed to identify the relationships between smoking, depression and the risk of pRBD (diagnosed by the RBD screening questionnaire) (15). These findings demonstrate the complexity and ongoing uncertainty that is encountered in the field of iRBD research.

Discrepancies between studies could be attributed to several factors. First, prior studies used traditional observational research designs, which are susceptible to confounding factors and reverse causation. Second, the inadequate sample sizes of some studies limit statistical power, increasing the likelihood of false-negative results and diminishing the generalizability of study conclusions. Finally, the low specificity of questionnaires used may have led to selection bias. Therefore, addressing limitations of prior studies and conducting further research using more robust study designs and larger sample sizes will be needed to obtain reliable and definitive conclusions.

Mendelian randomization (MR) is a statistical method that employs genetic variations as instrumental variables in the appraisal of causal associations between risk factors and particular diseases. The evidence level of MR is second only to randomized controlled trials (21–23). As genetic variations are randomly allocated to offspring through allelic randomization (24), results of MR studies are less likely to be affected by confounding factors and reverse causality, common limitations of traditional observational research (25). Furthermore, the genome-wide association study (GWAS) data on iRBD utilized in our MR analysis included a large sample, with each patient being diagnosed through PSG. This significantly enhances the credibility of our research findings.

Our study classified 29 potential modifiable risk factors into the following eight categories: anthropometric traits, metabolic traits, smoking, beverage consumption patterns, physical activity, education levels, mental illness, and sun exposure-related factors. We performed the MR approach to evaluate the causality between these factors and iRBD, providing more perspectives and evidence for screening and early intervention in at-risk populations.

## 2 Materials and methods

### 2.1 Study design

**Figure 1:**
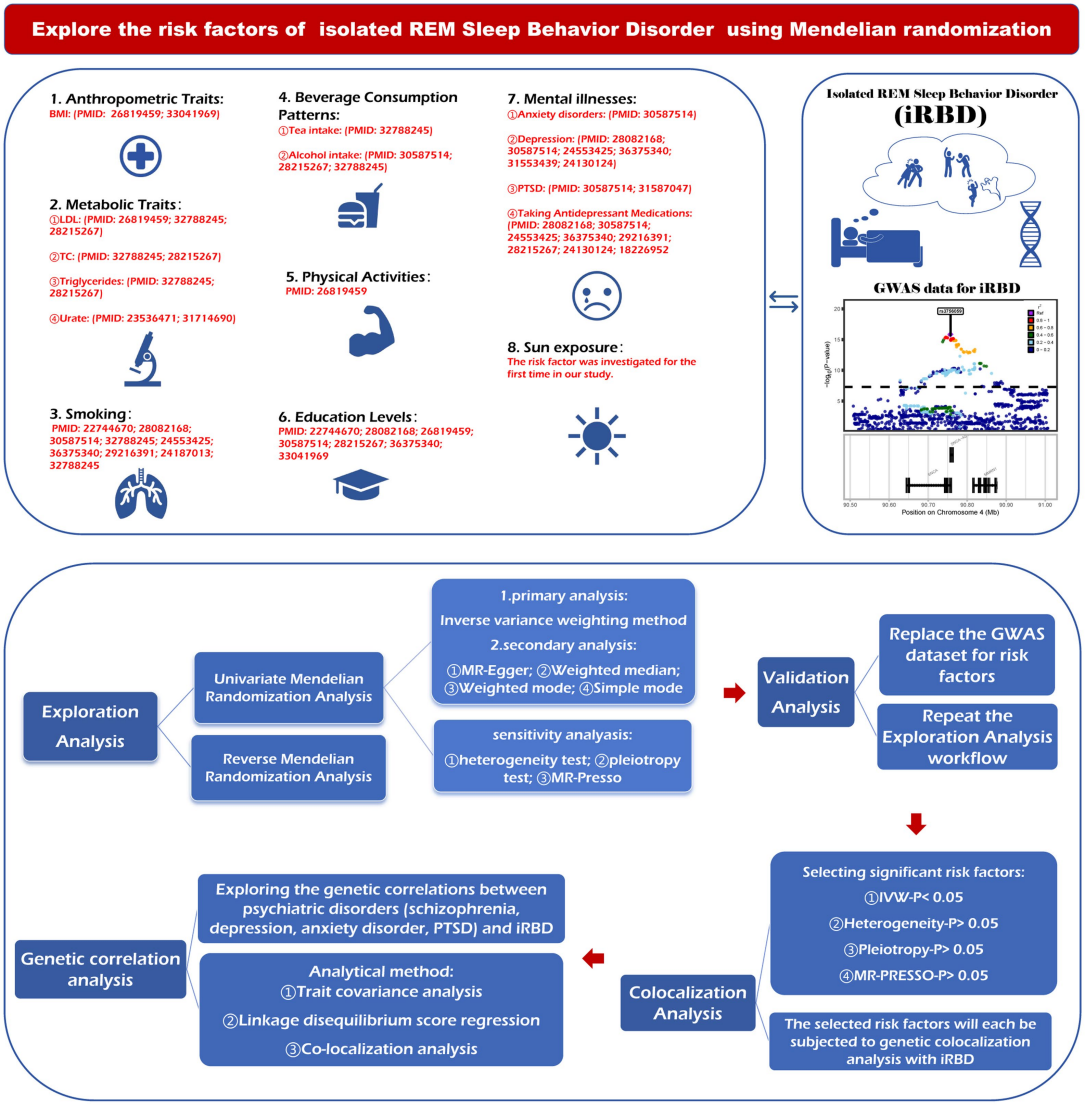
The figure outlines the steps of the research design. BMI, body mass index; LDL, low density lipoprotein; TC, total cholesterol; PTSD, post-traumatic stress disorder; MR, Mendelian randomization; GWAS, genome-wide association studies; MR-PRESSO, Mendelian randomization pleiotropy residual sun and outlier.

We conducted a systematic review of articles in the PubMed database to identify potential risk factors for iRBD. After selecting 29 modifiable factors, they were classified into the following eight categories: anthropometric traits, metabolic traits, smoking behavior, beverage consumption patterns, physical activity, education levels, mental illness, and sun exposure-related factors (Figure 1).

MR method employs genetic variations as instruments for exposure, so we assessed the causality between 29 modifiable factors and iRBD risk based on genetic variants strongly linked to these factors. The design of this MR study is illustrated in Figure 1. First, we conducted a two-sample MR analysis to evaluate the causality between these factors (as exposure) and iRBD (as outcome) in both the discovery and replication stages. Second, co-localization analyses of causal associations identified in MR study were performed to further explore whether the relationships depended on shared driver genes. Subsequently, reverse MR analysis was used to evaluate the possibility of reverse causality. Finally, since numerous previous studies have supported the connection between several common mental illness and iRBD; we explored their genetic correlations using trait covariance, linkage disequilibrium score regression (LDSC), and co-localization analyses.

MR research must satisfy the following three core assumptions: relevance, independence, and exclusivity. First, genetic variants should be highly correlated with an exposure. Second, genetic variation should not be associated with confounding factors. Lastly, genetic variants should not affect an outcome via a pathway other than that of the exposure (26, 27).

The GWAS summary datasets used in this study were derived from publicly available databases previously receiving ethical approval and all participants of each GWAS data provided informed consent. Our research strictly followed Strengthening the Reporting of Mendelian Randomization Studies (STROBE-MR) guidelines (28).

### 2.2 Data sources

The GWAS data for exposure factors in discovery and replication cohorts primarily originated from various datasets available on the IEU Open GWAS project website.^1^ Moreover, data on anxiety disorders in the discovery cohort; along with replication cohort data including urate levels, strenuous sports or other exercises, and years of schooling were sourced from the GWAS catalog.^2^ Data on PTSD (PMID:31594949) in the discovery cohort and schizophrenia (PMID:35396580) in the replication cohort were obtained from the Psychiatric Genomic Consortium (PGC).^3^ Detailed information is provided in Tables 1, 2.

**Table 1:** The risk factors for iRBD in the discovery phase.

Exposure	ID	NSNP	Sample	R^2^ (%)	F	Power	PMID or Consortium
Anthropometric traits							
Standing height	ukb-b-10787	724	461,950	8.55%	30.9	1.00	MRC-IEU
Weight	ukb-b-11842	470	461,632	5.01%	33.6	0.38	MRC-IEU
Body mass index	ukb-b-19953	426	461,460	5.33%	41.7	1.00	MRC-IEU
Basal metabolic rate	ukb-b-16446	517	454,874	3.51%	20.6	1.00	MRC-IEU
Trunk fat-free mass	ukb-a-292	394	331,030	3.38%	19.7	1.00	Neale Lab
Whole body fat-free mass	ukb-a-266	391	331,291	3.28%	19.1	1.00	Neale Lab
Trunk fat percentage	ukb-b-16407	365	454,613	3.66%	34	0.99	MRC-IEU
Whole body water mass	ukb-b-14540	527	454,888	3.36%	18.5	1.00	MRC-IEU
Metabolic traits							
HDL-C	ebi-a-GCST008035	15	17,751	>100%	9817.4	0.06	31217584
LDL-C	ebi-a-GCST90002412	290	431,167	11.00%	46.7	1.00	32493714
Total cholesterol	ieu-a-301	81	187,365	8.53%	75.1	0.15	GLGC
Triglycerides	ieu-b-111	267	441,016	8.24%	43.3	0.88	UK Biobank
Hypertension	ebi-a-GCST008036	7	21,936	2.47%	69.3	0.23	31217584
Urate levels	ebi-a-GCST001791	25	110,347	9.04%	123.3	0.99	23263486
Smoking							
Current tobacco smoking	ukb-a-16	9	337,030	0.04%	12.7	0.30	Neale Lab
Past tobacco smoking	ukb-b-2134	93	424,960	1.40%	56.5	1.00	MRC-IEU
Beverage consumption patters							
Coffee intake	ukb-b-5237	36	428,860	0.36%	21.3	1.00	MRC-IEU
Tea intake	ukb-b-6066	39	447,485	0.55%	37.1	1.00	MRC-IEU
Alcohol intake frequency	ukb-b-5779	94	462,346	2.32%	76.2	0.21	MRC-IEU
Physical activity							
Number of days/week of vigorous physical activity 10+ minutes	ukb-b-151	11	440,512	0.37%	136.9	1.00	MRC-IEU
Education levels							
Years of schooling	ieu-a-1239	299	766,345	2.14%	44.6	0.95	SSGAC
Mental illness							
Schizophrenia	ieu-b-5099	202	320,404	27.12%	201.2	1.00	PGC
Anxiety disorder	GWAS catalog: GCST90043712	46	456,348	1.86%	161.5	1.00	UK Biobank
Depression	GWAS catalog: ebi-a-GCST003769	11	180,866	0.20%	19.8	0.06	NA
PTSD	PMID:31594949	4	206,605	1.31%	551.5	1.00	PGC
Sun exposure-related factors							
Ease of sunburn	ukb-b-533	120	453,065	13.08%	51.4	1.00	MRC-IEU
Childhood sunburn occasions	ukb-b-13246	76	346,955	3.52%	36.9	1.00	MRC-IEU
Skin color	ukb-b-19560	135	456,692	5.20%	18.9	1.00	MRC-IEU
Use of sun/uv protection	ukb-b-7422	47	459,416	0.92%	29.2	1.00	MRC-IEU

NSNPs, number of single nucleotide polymorphism; R^2^, phenotype variance explained by genetics; F, F statistic; PMID, the publication ID in PubMed; HDL-C, High density lipoprotein cholesterol; LDL-C, Low-density lipoprotein cholesterol; PTSD, post-traumatic stress disorder; MRC-IEU, Medical Research Council Integrative Epidemiology Unit; GLGC, Global Lipids Genetics Consortium; SSGAC, the Social Science Genetic Association Consortium; PGC, Psychiatric Genomic Consortium; GIANT, Genetic Investigation of Anthropometric Traits; GSCAN, the GWAS and Sequencing Consortium of Alcohol and Nicotine use.

**Table 2:** The risk factors for iRBD in the replication phase.

Exposure	ID	NSNP	Sample	R^2^ (%)	F	Power	PMID or Consortium
Anthropometric traits							
Standing height	ieu-a-89	356	253,288	12.10%	59.2	1.00	GIANT
Weight	ieu-a-107	10	73,137	0.92%	64.3	0.82	GIANT
Body mass index	ieu-b-40	482	681,275	5.05%	52.7	1.00	GIANT
Metabolic traits							
HDL-C	ieu-b-109	295	403,943	8.24%	40.5	0.05	UK Biobank
LDL-C	ieu-b-110	141	440,546	5.42%	59.5	1.00	UK Biobank
Total cholesterol	met-c-933	20	21,491	8.46%	63	0.12	27005778
Triglycerides	ieu-a-302	53	177,861	5.44%	54.4	0.88	GLGC
Hypertension	finn-b-I9_HYPTENS	51	218,734	9.71%	371.6	0.77	NA
Urate levels	GWAS catalog: GCST90014015	226	389,404	4.09%	32.7	1.00	UK Biobank
Smoking							
Cigarettes smoked per day	ieu-b-142	20	249,752	3.31%	152.3	0.95	GSCAN
Beverage consumption patters							
Alcoholic drinks per week	ieu-b-73	30	335,394	0.61%	39.4	0.46	GSCAN
Physical activity							
Strenuous sports or other exercises	GWAS catalog: GCST006100	8	350,492	0.42%	109.3	0.31	UK Biobank
Education levels							
Years of schooling	GWAS catalog: GCST90029013	206	461,457	46.62%	853.5	0.97	UK Biobank
Mental illness							
Schizophrenia	NA	146	130,644	24.69%	188.8	0.06	35396580
Anxiety disorder	finn-b KRA_PSY_ANXIETY	5	218,792	0.70%	379.5	0.84	NA
Major Depressive Disorder	ieu-a-1187	30	480,359	1.28%	196.9	1.00	PGC
PTSD	finn-b-F5_PTSD	3	199,213	11.56%	6192.1	0.56	NA
Sun exposure-related factors							
Sunburn easily	finn-b-L12_NONIONRADISKIN	8	218,281	17.00%	377.8	1.00	NA
Phototoxic dermatitis	finn-b-L12_RADIATIONRELATEDSKIN	7	218,792	18.27%	2327.4	1.00	NA

Outcome data for iRBD were obtained from the International RBD Study Group involving approximately 9,447 individuals of European ancestry (1,061 cases and 8,386 controls) (29). This iRBD cohort included large cohorts of French, French Canadian, Italian and British origins, and smaller cohorts from different European populations. The cases were aged 68 +/− 9 years (standard deviation) on average and were 81% male, and the controls were aged 58.5 +/− 9 years on average, 68% male. Isolated RBD cases were diagnosed according to criteria outlined in the International Classification of Sleep Disorders (2nd or 3rd Edition), which includes video polysomnography findings. To ensure that case and control groups were comparable, principal components were employed to adjust for population substructure, taking factors such as sex and age into account.

### 2.3 Instrumental variable selection

A series of quality control measures were used to select appropriate instrumental variables. Single nucleotide polymorphisms (SNPs) were selected based on strong associations with the exposure (*p* < 5 × 10^−8^) and a minor allele frequency (MAF) > 0.01. To eliminate linkage disequilibrium (LD), SNPs were clumped based LD threshold (r^2^ < 0.001) and distance (10,000 kb). If SNPs were unavailable, high-LD proxies were selected for evaluation based on index SNP (r^2^ > 0. 8) per LD link or SNIPA guidelines (30, 31). Instrument strength was evaluated using the F-statistic, with an F-statistic >10 designated to mitigate potential bias arising from weak instruments (32, 33).

### 2.4 Statistical analysis

For the primary MR analysis, the inverse variance weighted (IVW) method (34) was used to evaluate causality. In addition, MR-Egger regression (35), weighted-median estimation (36) and weighted-mode (37) methods were used to supplement IVW findings. IVW analysis is sometimes susceptible to instrumental bias and multiple effects. Therefore, sensitivity analyses were used examine the validity and robustness of IVW results. Cochran’s Q statistic was used to assess heterogeneity among estimated IVW values. The MR-Egger intercept and MR Pleiotropy RESidual Sum and Outlier (MR-PRESSO) methods were used to detect horizontal pleiotropy. The MR-PRESSO method is useful for detecting outlier values. The analysis was conducted after all other MR analyses, after excluding aberrant SNPs (38). Leave-one-out sensitivity analysis was performed to validate the impact of each SNP loci.

All statistical analyses were performed using two-tailed Student’s t-tests. The effect estimates were presented as odds ratios (ORs) to more intuitively indicate the relationship between potential risk factors and iRBD. Values of *p* < 0. 05 were considered statistically significant. Finally, we interpreted findings based on statistical significance and consistency (via a comparison between discovery and validation cohorts). The mRnd was used to calculate the statistical power for Mendelian randomization.^4^ Statistical analyses were performed using the R statistical software (version 4. 2. 3) and relevant R packages.

### 2.5 Trait covariance, LDSC and co-localization analyses

The bivariate LDSC method was used to assess the genetic correlations between mental illness and iRBD (39–42). Trait covariance analysis was performed using the metaCCA package (43). We employed co-localization analysis using the Coloc R package (version 5.1.0.1) to further probe shared genetic underpinnings (44). The variant with the lowest value of p designated via MR analysis was most strongly associated with an exposure and selected as a reference. We included variants within 50 kb of the reference variant. The 1,000 Genomes v3 European ancestry dataset was used as an LD reference panel. In Bayesian co-localization analysis, five posterior probabilities are provided to determine whether two traits share the same variation. A posterior probability of hypothesis 4 greater than 0.8 indicates the presence of shared causal variants.

## 3 Results

Following the exclusion of SNPs in linkage disequilibrium, the number of SNPs analyzed in our study ranged from 3 to 724, with corresponding explained variances with diverse distributions ranging from 0.04 to 100% (Tables 1, 2). Notably, all included SNPs had F-statistics that surpassed the empirically determined threshold of 10, indicating the absence of any potential bias arising from weak instrumental variables, which confirms the credibility of our findings.

### 3.1 Results based on the discovery cohort

**Figure 2:**
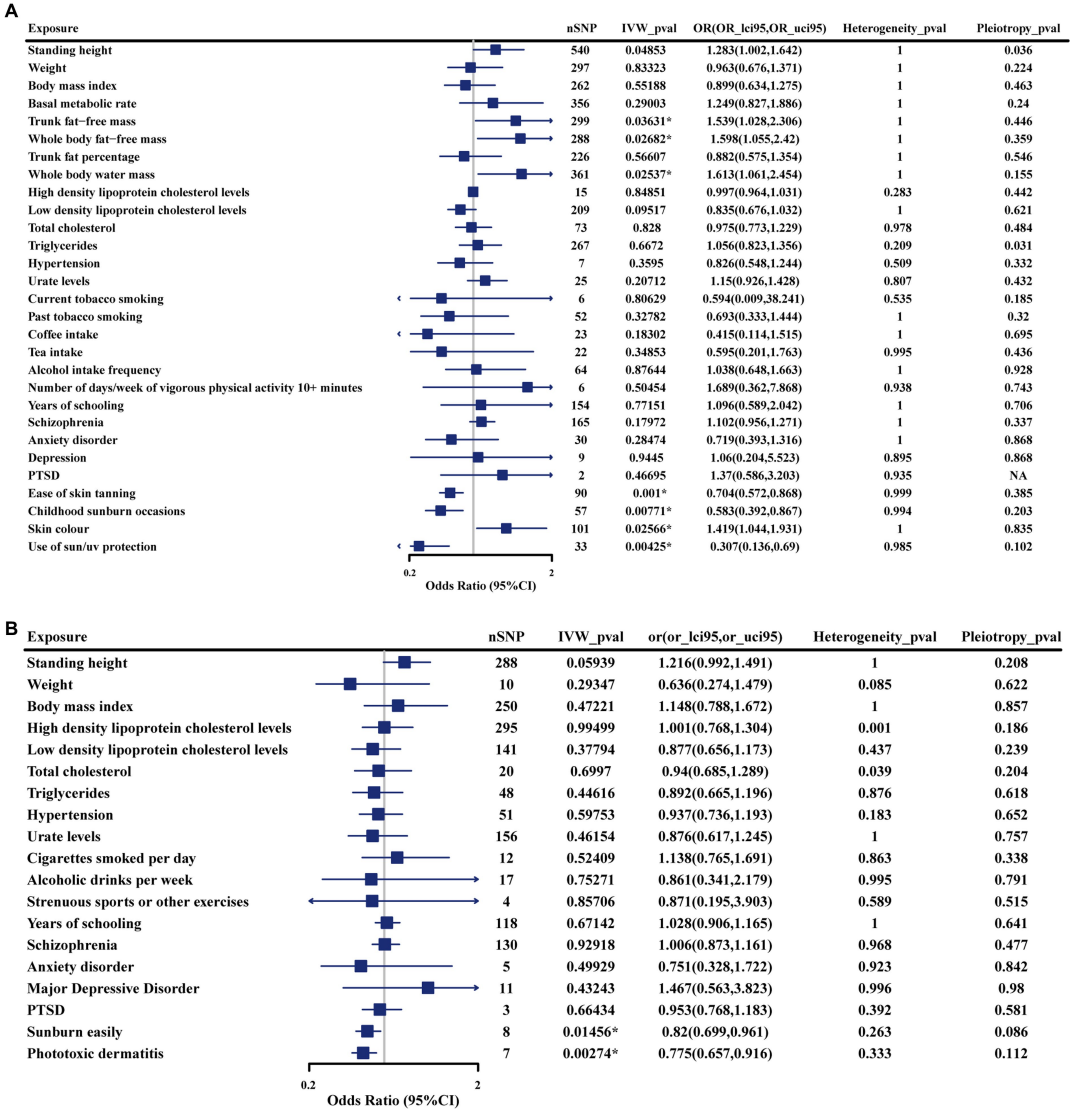
Forest plot illustrates the causal estimates of modifiable risk factors on iRBD using the inverse variance-weighted method. **(A)** Results from the discovery cohort. **(B)** Results from the replication cohort. SNP, singlenucleotide polymorphism; IVW: Inverse variance weighted; OR, odds ratio; 95%LCI, lower limit of 95% CI; 95%UCI, upper limit of 95% CI. Statistics significant. *p < 0.05.

In the discovery phase, we identified seven genetically determined factors across two categories that were causally associated with iRBD (Figure 2A). Their impacts on iRBD incidence were presented as odds ratios (ORs) with their corresponding 95% confidence intervals (95%CIs). Specifically, among anthropometric traits, trunk fat-free mass (OR: 1.54 [1.03, 2.31]; *p* = 0.036), whole-body fat-free mass (OR: 1.60 [1.06, 2.42]; *p* = 0.027), and whole-body water mass (OR: 1.61 [1.06, 2.45]; *p* = 0.025) emerged as risk factors. Within the category pertaining to sunlight exposure, factors such as ease of sunburn (OR: 0.70, 95% CI: 0.57–0.87; *p* = 0.001), childhood sunburn occasions (OR: 0.58, 95% CI: 0.39–0.87; *p* = 0.008), use of sun protection (OR: 0.31, 95% CI: 0.14–0.69; *p* = 0.004) were associated with a reduced risk of iRBD, while a deeper skin color (OR: 1.42, 95% CI: 1. 04–1. 93; *p* = 0.026) was linked to an increased incidence. Our study did not find evidence supporting causal associations between other factors and iRBD. Reverse MR analyses revealed that iRBD significantly increased drinking risk (Supplementary Figure S13).

Study conclusions were supported by weighted-median estimation, weighted-mode, and MR-Egger methods (Supplementary Table S2). Cochran’s Q statistic indicated no significant heterogeneity in SNP effects (*p* > 0.05). No evidence of potential horizontal pleiotropy was detected for the seven factors identified (*p* > 0. 05). To further assess the robustness of the results, we conducted MR-PRESSO tests on the included SNP loci. In addition, a leave-one-out sensitivity analysis was conducted to assess the influence of each SNP on the overall causal relationship. The results demonstrated that systematically removing individual SNPs and repeating the MR analysis did not reveal significant differences in the observed causal relationships (Supplementary Tables S3–S5).

### 3.2 Results based on the replication cohort

Sunburn easily (OR: 0.82 [0.70, 0.96]; *p* = 0.015) and phototoxic dermatitis (OR: 0.78 [0.66, 0.92]; *p* = 0.026) were confirmed to be protective factors for iRBD using replication phase data (Figure 2B). Furthermore, similar effect estimates were observed after applying weighted-median, weighted-mode, and MR-Egger methods (Supplementary Table S7). In sensitivity analyses, no heterogeneity or pleiotropy was observed, indicating the robustness of results (Supplementary Tables S8, S9). However, given that the data used regarding trunk and whole-body fat-free mass, and whole-body water mass were originated from a single source, we were unable to confirm the causal relationship between these factors and iRBD in the replication stage.

### 3.3 Co-localization analysis

**Figure 3:**
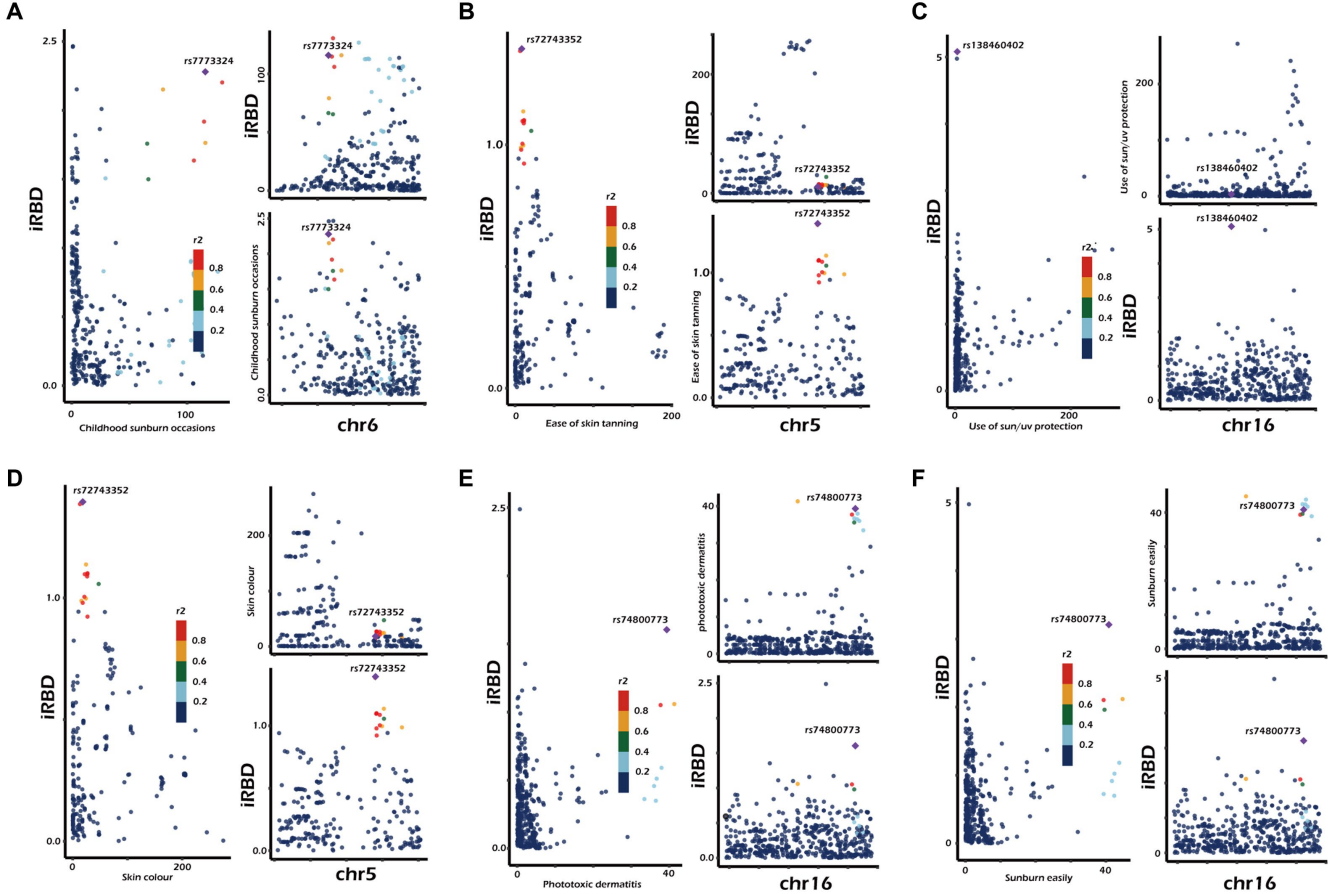
Genetic correlation of sun exposure-related factors with iRBD. Results were derived from co-localization analysis. chr, chromosome. The presented data are available in Supplementary Table 2.

To further investigate whether causal associations identified in discovery and validation phases were driven by shared genes, we conducted co-localization analysis. The results were as follows (Figure 3; Supplementary Table S17): ease of sunburn [coloc. Abf-posterior probability of hypothesis 4 (PPH4) = 0.057], childhood sunburn occasions (coloc. Abf-PPH4 = 0. 441), sun/ultraviolet (UV) protection (coloc. Abf-PPH4 = 0.426), and a deeper skin color (coloc. Abf-PPH4 = 0.039), sunburn easily (coloc. Abf-PPH4 = 0.301), and phototoxic dermatitis (coloc. Abf-PPH4 = 0.437). In general, a PPH4 exceeding 80% is considered indicative of robust colocalization evidence. However, in our research, the PPH4 values ranged from 3 to 44%. Additionally, there was no observed genetic overlap between three exposure factors (trunk fat-free mass, whole-body fat-free mass, and whole-body water mass) and iRBD occurring within a range of 50 kb of their respective lead SNPs.

### 3.4 Genetic correlation between common mental illness and iRBD

**Figure 4:**
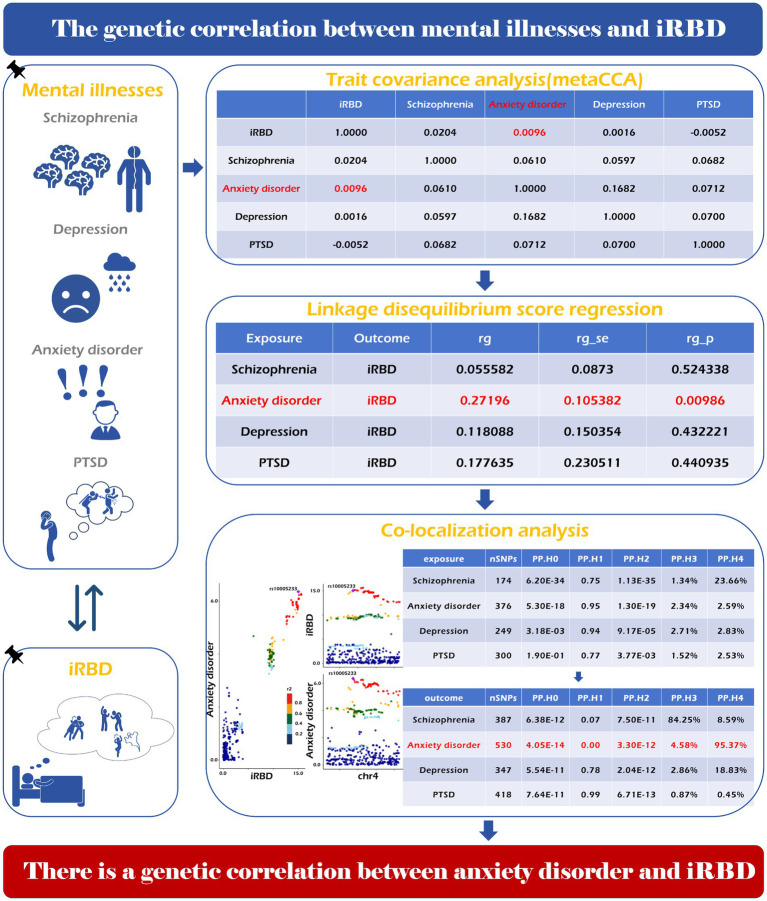
Genetic correlation of psychiatric disorders with iRBD. The specific analysis method, calculation process and final result have been shown in the picture.

In this study, we identified a genetic correlation between iRBD and anxiety disorders using the LDSC method (*r* = 0.2719, *p* = 0.0098) and trait covariance analysis (covariance = 0.0096). Moreover, co-localization analysis of iRBD (exposure) and anxiety disorders (outcome) identified a shared driver gene located within 50 kb of rs7822441 (Figure 4). However, no evidence supported a genetic correlation between schizophrenia, depression, PTSD, and iRBD.

## 4 Discussion

Considering the prevailing ambiguity surrounding risk factors for iRBD, our investigation marks the pioneering utilization of MR analysis to systematically assess the causality between 29 potential determinants and iRBD. Our study identified a causal relationship between sunlight exposure the incidence of iRBD. Specifically, utilizing sun protection, and ease of sunburn, childhood sunburn occasions, and phototoxic dermatitis may reduce risk of iRBD. In contrast, individuals with a deep skin color are at increased iRBD risk. Furthermore, our research revealed no causal associations between smoking, coffee consumption, alcohol intake, educational attainment, mental illness, and other risk factors previously identified through observational studies and iRBD. A genetic correlation between anxiety disorders and iRBD was observed.

Previous researches have demonstrated that increasing exposure to sunlight can diminish risk of PD by elevating levels of 25-hydroxyvitamin D (10, 11, 45–47). IRBD, as a prodromal stage of α-synucleinopathies, is also the strongest predictor of PD onset. However, there is currently a lack of research exploring the relationship between sunlight exposure and iRBD. Therefore, we selected several factors related to sun exposure to investigate whether they were causally associated with iRBD. Surprisingly, we found that individuals who were prone to sunburn, had a childhood history of sunburn and phototoxic dermatitis, and took sun protection measures tended to have a reduced risk of developing iRBD. Conversely, individuals with a deep skin color were at increased risk of developing the disease. At first glance, these results seem to contradict each other. However, upon closer examination, intriguing connections emerge.

**Figure 5:**
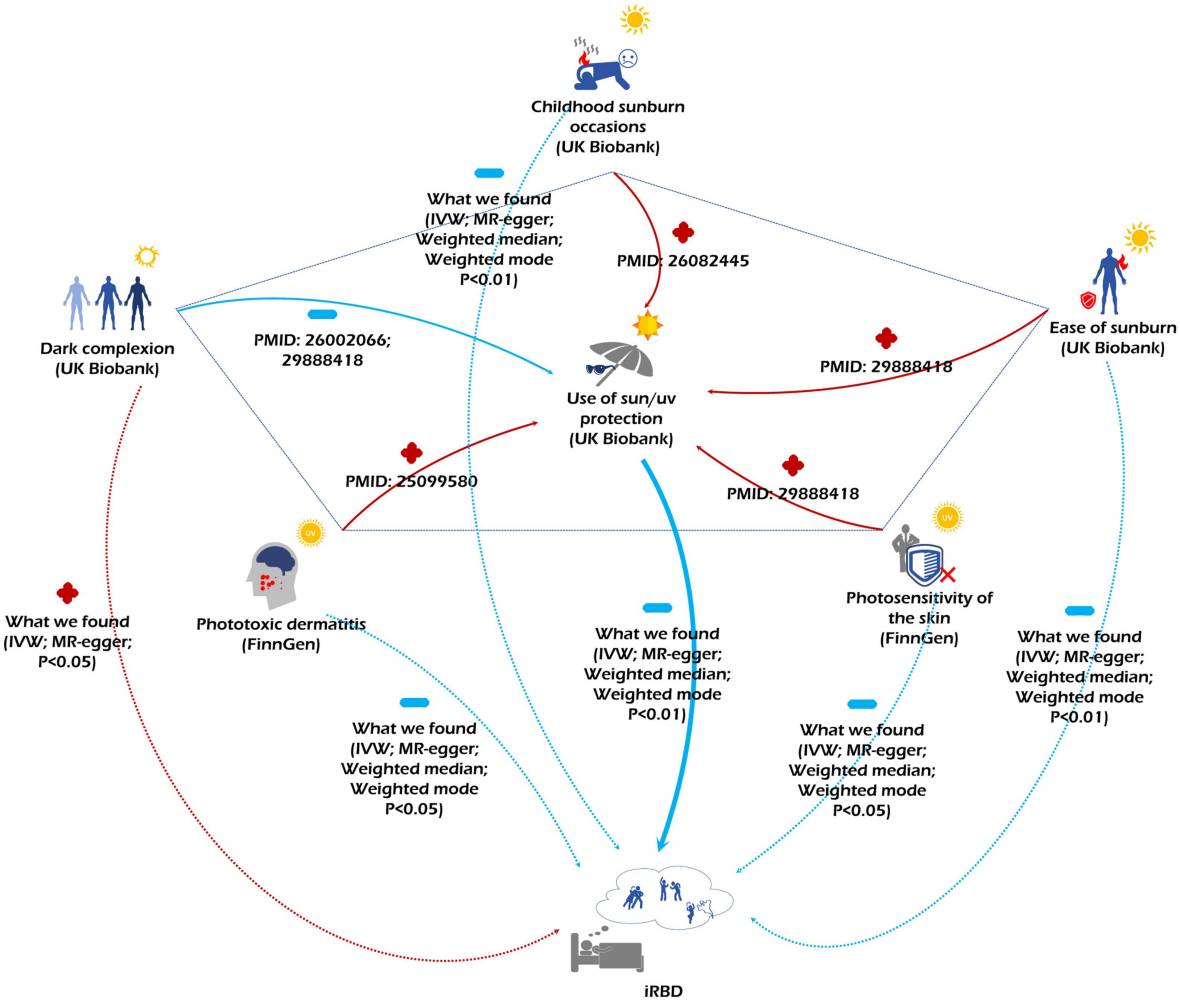
The figure depicts the relationships among various sun exposure-related factors and their associated risks for iRBD. Red arrows accompanied by a “+” indicate a promoting effect, while blue arrows paired with a “-” signify an inhibitory effect.

In our discovery cohort, four consistent MR methods initially demonstrated that sun protection measures decrease iRBD incidence, underscoring the reliability of our findings. It is well-documented that personal protective behaviors play a significant role in moderating UV exposure (48), suggesting that excessive sun exposure could pose a risk for iRBD. Deep skin color emerged as an iRBD risk factor, a conclusion reinforced by both IVW and MR-Egger methods. Prior researches have indicated that individuals with deeper skin tones often exhibit reduced sunlight sensitivity, possibly leading to less frequent use of sun protection and heightened sun exposure (49–53). Data from both our discovery and replication cohorts, sourced from FinnGen and the UK Biobank, revealed that those more prone to sunburn experience a lower iRBD incidence. According to Fitzpatrick’s photo-type classification, sunburn-susceptible individuals are viewed as UV-sensitive and typically limit their sun exposure (48, 54–58). Moreover, our research identified childhood sunburn history and photosensitive dermatitis as protective factors against iRBD. As phototoxic dermatitis is UV radiation-related, those afflicted often adopt enhanced sun protection, thereby reducing their overall sun exposure relative to the general population (59). Earlier studies also highlight that childhood sunburn experiences can prompt increased sun protection use (60). Collectively, our data indicate that excessive sun exposure elevates iRBD risk, but vigilant sun protection can diminish this risk (Figure 5). This insight is notable, especially given past findings linking farmers, who typically have heightened sunlight exposure (61–63) with a higher iRBD risk (13, 16, 17, 20). Identifying this ubiquitous and modifiable iRBD risk factor carries profound public health significance.

Sunlight exposure has opposite effects on the incidence rates of iRBD and PD, indicating that despite the tendency of iRBD patients to progress to PD, there are still differences in the pathogenesis of the two diseases. The decline in melatonin levels may explain the increased incidence of iRBD caused by excessive sun exposure. Melatonin, known as the chemical expression of darkness, is a sunlight-dependent indole compound primarily released by the pineal gland, which is crucial for regulating the human biological clock and sleep cycle (64, 65). Sun exposure significantly influences melatonin levels. Previous observational studies have noted that during sunnier summer months, melatonin levels are lower and last for shorter periods compared to winter (66, 67). From the poles to the equator, with increasing sunlight intensity and duration, melatonin secretion decreases (68, 69). This phenomenon is biochemically explained by excessive sunlight suppressing the activity of key enzymes in the synthesis pathway (70, 71), while also activating melanopsin produced by retinal ganglion cells, thereby suppressing the synthesis and release of melatonin in the pineal gland (70, 72, 73). Experimental researches have also confirmed the negative correlation between light exposure duration and intensity and melatonin production (74–78).

Melatonin might have a protective effect against the onset of iRBD. Firstly, impaired glycine and GABA neurotransmission could be potential mechanisms underlying iRBD (79–81). Studies have demonstrated that melatonin can potentiate the action of GABA on GABA_A_ receptors located on motoneurons, directly augmenting tonic GABA_A_ transmission (80, 82, 83). This action ultimately triggers REM sleep atonia, thereby improving iRBD symptoms. Secondly, the α-synuclein accumulation in the brainstem is also a mechanism contributing to the pathogenesis of iRBD. Melatonin can reduce the aggregation of α-synuclein and exerts neuroprotective effects by scavenging free radicals (84, 85), stimulating glutathione synthesis (86, 87), enhancing antioxidant enzyme synthesis, and inhibiting the production of pro-oxidant enzymes (88, 89), thus alleviating α-synuclein’s mitochondrial toxicity (90, 91). Thirdly, studies have reported that inflammation plays a role in the pathogenesis of iRBD (92, 93). Melatonin can exert anti-inflammatory effects by acting as an antioxidant, modulating the expression of inducible nitric oxide synthase, and influencing inflammatory signaling pathways and the production of inflammation-related cytokines (94–97). Finally, previous case–control and randomized controlled trials have confirmed that melatonin can improve symptoms of RBD (75, 98–107). Thus, excessive sun exposure may lead to melatonin levels reduction in patients, consequently diminishing its neuroprotective, antioxidant, and anti-inflammatory effects. This could potentially explain the correlation between increased sun exposure and a higher incidence of iRBD.

A community-based study demonstrated that having a low BMI may increase risk of iRBD (17). This study did not identify a causal relationship between BMI and iRBD risk. However, we found that increased trunk and whole-body fat-free mass were associated with in increased risk of iRBD. Compared to BMI, Fat-free mass, which represents trunk non-adipose tissue mass (trunk mass after subtracting the mass of fat tissue), provides an accurate depiction of body composition and serves as a valuable diagnostic tool for obesity (108–110). Although the mechanisms underlying these indicators remain unclear, they offer valuable guidance for identifying populations at high risk of developing iRBD.

Previous studies of the relationship between lifestyle factors (such as smoking and alcohol consumption) and iRBD have been fraught with contradictions and controversies. Studies have suggested that smoking (13, 14, 19, 20, 111, 112), alcohol consumption (19, 20), and tea consumption (20) increase risk of iRBD. However, other researches have failed to establish causal associations between the factors and iRBD (15–17). Our study did not support the notion that smoking, coffee consumption, or tea consumption are risk factors for iRBD. With a statistical power of 1, these findings are highly robust. Inconsistency in results of observational studies may arise from the possible presence of confounding factors such as socioeconomic status, which may have obscured the true relationship between lifestyle factors and iRBD. Furthermore, our reverse MR analysis indicated that iRBD increased alcohol consumption, suggesting that the relationship between alcohol use and iRBD that was identified in previous observational studies may have been due to reverse causation.

Some studies have suggested that individuals low education levels are at increased risk of iRBD development (13, 15, 18, 113). However, in our study, we did not find a causal association between years of schooling and iRBD. Socioeconomic status can influence educational experiences, living conditions, and medical services accessed by individuals. These factors may affect the incidence of various diseases. This suggests that socioeconomic status may confound the relationship between education and iRBD in observational studies. This was supported by findings of a CLSA population-based cohort study that demonstrated that the association between education and iRBD was diminished after adjusting for socioeconomic level (19). In addition, we should not overlook the influence of education levels on a patient’s willingness to seek medical care, a phenomenon that may contribute to selection bias in observational studies.

Previous studies have reported a close association between some mental illnesses and iRBD, indicating that anxiety, depression, psychological stress, and PTSD may be risk factors for iRBD (13, 14, 19, 113–116). Nevertheless, we found no causality between mental illness and iRBD in both discovery and replication phases of our study. The discrepancies between prior studies and our findings can be elucidated from several perspectives: (1) overexposure: individuals with mental illness are more likely to report sleep problems (117, 118), leading to an increased frequency of sleep evaluations, which could inflate the detection rate of sleep disorders. Such overexposure may result in an overestimation of the association between mental illness and iRBD. (2) Misdiagnosis: depression and anxiety disorders often coexist with obstructive sleep apnea (OSA) (119, 120), a condition that shares many clinical presentations with iRBD. Distinguishing between the two without PSG is challenging (2). Observational studies using scales to screen for iRBD patients may overstate its association with mental illness. (3) Genetic Correlation: our research identified a genetic correlation between anxiety disorders and iRBD. This makes the concurrent occurrence of both conditions more probable, potentially obscuring their genuine relationship. It is worth noting that while our study does not support a causal relationship between depression and iRBD, the limited statistical power (0.06) of the instrumental variable for depression may lead to false-negative results. Further studies are needed to better understand the link between depression and iRBD. Conducting additional family-based, genetic association, and molecular genetic studies are essential for exploring genetic links between mental illness and iRBD.

Our MR study had several strengths. First, it is the first MR study to investigate modifiable risk factors associated with iRBD, aiming to substantially reduce bias due to the presence of confounding factors and reverse causality commonly encountered in observational studies. Second, to evaluate the relationships between exposure factors and iRBD, we utilized the most extensive GWAS datasets available. In both the discovery and replication stages, separate exposure datasets were leveraged to ensure result consistency. Our analysis incorporated a range of methods, including MR, LDSC, trait covariance, and colocalization analyses, to probe potential associations. When MR failed to pinpoint a connection, we assessed the power to ensure result reliability. These strategies significantly bolstered the reliability and validity of our conclusions. Third, our findings revealed that excessive sun exposure increases the risk of iRBD, while sun protection acts as a potent preventive measure. Identifying this prevalent and modifiable risk factor in daily life is pivotal for devising effective preventive strategies, holding substantial public health significance.

Despite its strengths, our study has several limitations. First, our study showed discrepancies with past observational studies, possibly due to potential confounding bias and reverse causality in previous researches, or our limited iRBD data sample size, restricting the wide applicability of our findings. Thus, we advocate for stricter observational study designs and the application of larger GWAS data in MR analyses to clarify these issues more distinctly. Second, although our discovery cohort identified potential causality among trunk fat-free mass, whole-body fat-free mass, whole-body water mass and iRBD, the GWAS data for these exposure factors were exclusively sourced from the UK Biobank. Future research utilizing different datasets is warranted to validate our findings. Third, we identified the causal associations between sun exposure-related factors and iRBD using MR analysis. However, further observational studies will be needed to confirm our findings. Furthermore, as the statistical power of the depression data is insufficient, higher quality GWAS data are required. In addition, there is a lack of GWAS datasets on antipsychotic drugs to further investigate the causality between antipsychotic medications and iRBD.

## 5 Conclusion

In this study, we discovered that excessive sun exposure increase the risk of iRBD. However, Our research does not corroborate the findings from previous observational studies that identified alcohol consumption, smoking, mental illness, and low education levels as risk factors for iRBD. Interestingly, we did observe a genetic correlation between anxiety disorders and iRBD. These insights offer fresh perspectives for screening high-risk populations and devising preventive measures.

## Data availability statement

The original contributions presented in the study are included in the article/Supplementary material, further inquiries can be directed to the corresponding author.

## Ethics statement

Ethical review and approval was not required for the study on human participants in accordance with the local legislation and institutional requirements. Written informed consent from the patients was not required to participate in this study in accordance with the national legislation and the institutional requirements.

## Author contributions

R-YZ: Conceptualization, Formal analysis, Methodology, Validation, Visualization, Writing – original draft, Writing – review & editing. F-JL: Conceptualization, Data curation, Software, Supervision, Validation, Visualization, Writing – review & editing. QZ: Investigation, Resources, Writing – review & editing. L-HX: Software, Writing – review & editing. J-YZ: Software, Writing – review & editing. JZ: Funding acquisition, Project administration, Supervision, Validation, Writing – review & editing.

## Footnotes

^1^https://gwas.mrcieu.ac.uk

^2^https://www.ebi.ac.uk/gwas

^3^https://pgc.unc.edu

^4^https://cnsgenomics.shinyapps.io/mRnd/

## References

[1] Schenck CH Bundlie SR Ettinger MG Mahowald MW. (1986). Chronic behavioral disorders of human REM sleep: a new category of parasomnia. Sleep 9, 293–308. doi: 10.1093/sleep/9.2.293 3505730

[2] Iranzo A Santamaria J Tolosa E. Idiopathic rapid eye movement sleep behaviour disorder: diagnosis, management, and the need for neuroprotective interventions. Lancet Neurol. (2016) 15:405–19. doi: 10.1016/S1474-4422(16)00057-0 26971662

[3] Olson EJ Boeve BF Silber MH. Rapid eye movement sleep behaviour disorder: demographic, clinical and laboratory findings in 93 cases. Brain. (2000) 123:331–339. doi: 10.1093/brain/123.2.331 10648440

[4] Boeve BF. REM sleep behavior disorder: Updated review of the core features, the REM sleep behavior disorder-neurodegenerative disease association, evolving concepts, controversies, and future directions. Ann N Y Acad Sci. (2010) 1184:15–54. doi: 10.1111/j.1749-6632.2009.05115.x 20146689 PMC2902006

[5] Dauvilliers Y Schenck CH Postuma RB Iranzo A Luppi PH Plazzi G. REM sleep behaviour disorder. Nat Rev Dis Primers (2018) 4:19. doi: 10.1038/s41572-018-0016-5 30166532

[6] Postuma RB Gagnon JF Vendette M Desjardins C Montplaisir JY. Olfaction and color vision identify impending neurodegeneration in rapid eye movement sleep behavior disorder. Ann Neurol. (2011) 69:811–818. doi: 10.1002/ana.22282 21246603

[7] Iranzo A Tolosa E Gelpi E Molinuevo JL Valldeoriola F Serradell M. Neurodegenerative disease status and post-mortem pathology in idiopathic rapid-eye-movement sleep behaviour disorder: an observational cohort study. Lancet Neurol. (2013) 12:443–453. doi: 10.1016/S1474-4422(13)70056-5 23562390

[8] Schenck CH Boeve BF Mahowald MW. Delayed emergence of a parkinsonian disorder or dementia in 81% of older men initially diagnosed with idiopathic rapid eye movement sleep behavior disorder: a 16-year update on a previously reported series. Sleep Med. (2013) 14:744–748. doi: 10.1016/j.sleep.2012.10.009 23347909

[9] Miglis MG Adler CH Antelmi E Arnaldi D Baldelli L Boeve BF. Biomarkers of conversion to alpha-synucleinopathy in isolated rapid-eye-movement sleep behaviour disorder. Lancet Neurol. (2021) 20:671–684. doi: 10.1016/S1474-4422(21)00176-9 34302789 PMC8600613

[10] Wang J Yang D Yu Y Shao G Wang Q. Vitamin D and sunlight exposure in newly-diagnosed Parkinson's disease. Nutrients. (2016) 8:142. doi: 10.3390/nu8030142, PMID: 26959053 PMC4808871

[11] Zhou Z Zhou R Zhang Z Li K. The association between vitamin D status, vitamin D supplementation, sunlight exposure, and Parkinson's disease: a systematic review and meta-analysis. Med Sci Monit. (2019) 25:666–74. doi: 10.12659/msm.912840, PMID: 30672512 PMC6352758

[12] Booij J Tellier SP Seibyl J Vriend C. Dopamine Transporter Availability in Early Parkinson’s Disease is Dependent on Sunlight Exposure. Mov Disord. (2023) 38:2131–2135. doi: 10.1002/mds.29597 37670567

[13] Arnaldi D Antelmi E St Louis EK Postuma RB Arnulf I. Idiopathic REM sleep behavior disorder and neurodegenerative risk: to tell or not to tell to the patient? How to minimize the risk? Sleep Med Rev. (2017) 36:82–95. doi: 10.1016/j.smrv.2016.11.002, PMID: 28082168

[14] Frauscher B Jennum P Ju YE Postuma RB Arnulf I Cochen De Cock V. Comorbidity and medication in REM sleep behavior disorder: a multicenter case-control study. Neurology. (2014) 82:1076–9. doi: 10.1212/wnl.0000000000000247, PMID: 24553425 PMC3962997

[15] Ma JF Qiao Y Gao X Liang L Liu XL Li DH. A community-based study of risk factors for probable rapid eye movement sleep behavior disorder. Sleep Med. (2017) 30:71–6. doi: 10.1016/j.sleep.2016.06.027, PMID: 28215267

[16] Postuma RB Montplaisir JY Pelletier A Dauvilliers Y Oertel W Iranzo A. Environmental risk factors for REM sleep behavior disorder: a multicenter case-control study. Neurology. (2012) 79:428–34. doi: 10.1212/WNL.0b013e31825dd383, PMID: 22744670 PMC3405255

[17] Wong JC Li J Pavlova M Chen S Wu A Wu S. Risk factors for probable REM sleep behavior disorder: a community-based study. Neurology. (2016) 86:1306–12. doi: 10.1212/wnl.0000000000002414, PMID: 26819459 PMC4826335

[18] Wu Z Wu J Xie C Wang L Li H Zhang M. Risk factors for rapid eye-movement sleep-related behavioral disorders (RBDs): a systematic review and a meta-analysis. Gen Hosp Psychiatry. (2022) 79:118–27. doi: 10.1016/j.genhosppsych.2022.10.009, PMID: 36375340

[19] Yao C Fereshtehnejad SM Keezer MR Wolfson C Pelletier A Postuma RB. Risk factors for possible REM sleep behavior disorder: a CLSA population-based cohort study. Neurology. (2018) 92:e475–85. doi: 10.1212/wnl.0000000000006849, PMID: 30587514 PMC6369902

[20] Zhang H Gu Z Yao C Cai Y Li Y Mao W. Risk factors for possible REM sleep behavior disorders: a community-based study in Beijing. Neurology. (2020) 95:e2214–24. doi: 10.1212/wnl.0000000000010610, PMID: 32788245 PMC7713784

[21] Katan MB. Commentary: Mendelian Randomization, 18 years on. Int J Epidemiol. (2004) 33:10–11. doi: 10.1093/ije/dyh023 15075137

[22] Sekula P Del Greco MF Pattaro C Kottgen A. Mendelian Randomization as an Approach to Assess Causality Using Observational Data. J Am Soc Nephrol. (2016) 27:3253–3265. doi: 10.1681/ASN.2016010098 27486138 PMC5084898

[23] Larsson SC Burgess S Michaelsson K. Association of Genetic Variants Related to Serum Calcium Levels With Coronary Artery Disease and Myocardial Infarction. JAMA. (2017) 318:371–380. doi: 10.1001/jama.2017.8981 28742912 PMC5817597

[24] Lawlor DA Harbord RM Sterne JA Timpson N Davey Smith G. Mendelian randomization: using genes as instruments for making causal inferences in epidemiology. Stat Med. (2008) 27:1133–1163. doi: 10.1002/sim.3034 17886233

[25] Smith GD Lawlor DA Harbord R Timpson N Day I Ebrahim S. Clustered environments and randomized genes: a fundamental distinction between conventional and genetic epidemiology. PLoS Med. (2007) 4:e352. doi: 10.1371/journal.pmed.0040352 18076282 PMC2121108

[26] Hemani G Zheng J Elsworth B Wade KH Haberland V Baird D. The MR-base platform supports systematic causal inference across the human phenome. Elife. (2018) 7:e34408. doi: 10.7554/eLife.34408, PMID: 29846171 PMC5976434

[27] Yavorska OO Burgess S. MendelianRandomization: an R package for performing mendelian randomization analyses using summarized data. Int J Epidemiol. (2017) 46:1734–9. doi: 10.1093/ije/dyx034, PMID: 28398548 PMC5510723

[28] Skrivankova VW Richmond RC Woolf BAR Yarmolinsky J Davies NM Swanson SA. Strengthening the reporting of observational studies in epidemiology using mendelian randomization: the STROBE-MR statement. JAMA. (2021) 326:1614–21. doi: 10.1001/jama.2021.18236, PMID: 34698778

[29] Krohn L Heilbron K Blauwendraat C Reynolds RH Yu E Senkevich K. Genome-wide association study of REM sleep behavior disorder identifies polygenic risk and brain expression effects. Nat Commun. (2022) 13:7496. doi: 10.1038/s41467-022-34732-5, PMID: 36470867 PMC9722930

[30] Arnold M Raffler J Pfeufer A Suhre K Kastenmüller G. SNiPA: an interactive, genetic variant-centered annotation browser. Bioinformatics. (2015) 31:1334–6. doi: 10.1093/bioinformatics/btu779, PMID: 25431330 PMC4393511

[31] Machiela MJ Chanock SJ. LDlink: a web-based application for exploring population-specific haplotype structure and linking correlated alleles of possible functional variants. Bioinformatics. (2015) 31:3555–7. doi: 10.1093/bioinformatics/btv402, PMID: 26139635 PMC4626747

[32] Palmer TM Lawlor DA Harbord RM Sheehan NA Tobias JH Timpson NJ. Using multiple genetic variants as instrumental variables for modifiable risk factors. Stat Methods Med Res. (2012) 21:223–42. doi: 10.1177/0962280210394459, PMID: 21216802 PMC3917707

[33] Pierce BL Ahsan H Vanderweele TJ. Power and instrument strength requirements for mendelian randomization studies using multiple genetic variants. Int J Epidemiol. (2011) 40:740–52. doi: 10.1093/ije/dyq151, PMID: 20813862 PMC3147064

[34] Burgess S Bowden J Fall T Ingelsson E Thompson SG. Sensitivity analyses for robust causal inference from mendelian randomization analyses with multiple genetic variants. Epidemiology. (2017) 28:30–42. doi: 10.1097/ede.0000000000000559 27749700 PMC5133381

[35] Bowden J Davey Smith G Burgess S. Mendelian randomization with invalid instruments: effect estimation and bias detection through Egger regression. Int J Epidemiol. (2015) 44:512–25. doi: 10.1093/ije/dyv080, PMID: 26050253 PMC4469799

[36] Bowden J Davey Smith G Haycock PC Burgess S. Consistent estimation in mendelian randomization with some invalid instruments using a weighted median estimator. Genet Epidemiol. (2016) 40:304–14. doi: 10.1002/gepi.21965, PMID: 27061298 PMC4849733

[37] Hartwig FP Davey Smith G Bowden J. Robust inference in summary data mendelian randomization via the zero modal pleiotropy assumption. Int J Epidemiol. (2017) 46:1985–98. doi: 10.1093/ije/dyx102, PMID: 29040600 PMC5837715

[38] Verbanck M Chen CY Neale B Do R. Detection of widespread horizontal pleiotropy in causal relationships inferred from mendelian randomization between complex traits and diseases. Nat Genet. (2018) 50:693–8. doi: 10.1038/s41588-018-0099-7, PMID: 29686387 PMC6083837

[39] Bulik-Sullivan B Finucane HK Anttila V Gusev A Day FR Loh PR. An atlas of genetic correlations across human diseases and traits. Nat Genet. (2015) 47:1236–41. doi: 10.1038/ng.3406, PMID: 26414676 PMC4797329

[40] Bulik-Sullivan BK Loh PR Finucane HK Ripke S Yang J Patterson N. LD score regression distinguishes confounding from polygenicity in genome-wide association studies. Nat Genet. (2015) 47:291–5. doi: 10.1038/ng.3211, PMID: 25642630 PMC4495769

[41] Gazal S Finucane HK Furlotte NA Loh PR Palamara PF Liu X. Linkage disequilibrium-dependent architecture of human complex traits shows action of negative selection. Nat Genet. (2017) 49:1421–7. doi: 10.1038/ng.3954, PMID: 28892061 PMC6133304

[42] Zheng J Erzurumluoglu AM Elsworth BL Kemp JP Howe L Haycock PC. LD hub: a centralized database and web interface to perform LD score regression that maximizes the potential of summary level GWAS data for SNP heritability and genetic correlation analysis. Bioinformatics. (2017) 33:272–9. doi: 10.1093/bioinformatics/btw613, PMID: 27663502 PMC5542030

[43] Cichonska A Rousu J Marttinen P Kangas AJ Soininen P Lehtimäki T. metaCCA: summary statistics-based multivariate meta-analysis of genome-wide association studies using canonical correlation analysis. Bioinformatics. (2016) 32:1981–9. doi: 10.1093/bioinformatics/btw052, PMID: 27153689 PMC4920109

[44] Wallace C. A more accurate method for colocalisation analysis allowing for multiple causal variants. PLoS Genet. (2021) 17:e1009440. doi: 10.1371/journal.pgen.1009440, PMID: 34587156 PMC8504726

[45] Koduah P Paul F Dörr JM. Vitamin D in the prevention, prediction and treatment of neurodegenerative and neuroinflammatory diseases. EPMA J. (2017) 8:313–25. doi: 10.1007/s13167-017-0120-8, PMID: 29209434 PMC5700019

[46] Mpandzou G Aït Ben Haddou E Regragui W Benomar A Yahyaoui M. Vitamin D deficiency and its role in neurological conditions: a review. Rev Neurol (Paris). (2016) 172:109–22. doi: 10.1016/j.neurol.2015.11.005, PMID: 26867662

[47] Wang L Evatt ML Maldonado LG Perry WR Ritchie JC Beecham GW. Vitamin D from different sources is inversely associated with Parkinson disease. Mov Disord. (2015) 30:560–6. doi: 10.1002/mds.26117, PMID: 25545356 PMC4390412

[48] Modenese A Bisegna F Borra M Grandi C Gugliermetti F Militello A. Outdoor work and solar radiation exposure: evaluation method for epidemiological studies. Med Pr. (2016) 67:577–87. doi: 10.13075/mp.5893.00461, PMID: 27819697

[49] Buller DB Cokkinides V Hall HI Hartman AM Saraiya M Miller E. Prevalence of sunburn, sun protection, and indoor tanning behaviors among Americans: review from national surveys and case studies of 3 states. J Am Acad Dermatol. (2011) 65:S114.e1–S114.e11. doi: 10.1016/j.jaad.2011.05.033 22018060

[50] Calderón TA Bleakley A Jordan AB Lazovich D Glanz K. Correlates of sun protection behaviors in racially and ethnically diverse U.S. adults. Prev Med Rep. (2019) 13:346–53. doi: 10.1016/j.pmedr.2018.12.006, PMID: 30792951 PMC6369227

[51] Holman DM Berkowitz Z Guy GP Jr Hawkins NA Saraiya M Watson M. Patterns of sunscreen use on the face and other exposed skin among US adults. J Am Acad Dermatol. (2015) 73:83–92.e1. doi: 10.1016/j.jaad.2015.02.1112, PMID: 26002066 PMC4475428

[52] Martin A Liu J Thatiparthi A Ge S Wu JJ. Asian Americans are less likely to wear sunscreen compared with non-Hispanic whites. J Am Acad Dermatol. (2022) 86:167–9. doi: 10.1016/j.jaad.2020.12.079, PMID: 33715929

[53] Summers P Bena J Arrigain S Alexis AF Cooper K Bordeaux JS. Sunscreen use: non-Hispanic blacks compared with other racial and/or ethnic groups. Arch Dermatol. (2011) 147:863–4. doi: 10.1001/archdermatol.2011.172, PMID: 21768492 PMC4112119

[54] Armstrong BK. Epidemiology of malignant melanoma: intermittent or total accumulated exposure to the sun? J Dermatol Surg Oncol. (1988) 14:835–49. doi: 10.1111/j.1524-4725.1988.tb03588.x 3397443

[55] Eilers S Bach DQ Gaber R Blatt H Guevara Y Nitsche K. Accuracy of self-report in assessing Fitzpatrick skin phototypes I through VI. JAMA Dermatol. (2013) 149:1289–94. doi: 10.1001/jamadermatol.2013.6101, PMID: 24048361

[56] Gutierrez EL Galarza C Ramos W Tello M Jiménez G Ronceros G. Skin diseases in the Peruvian Amazonia. Int J Dermatol. (2010) 49:794–800. doi: 10.1111/j.1365-4632.2010.04473.x, PMID: 20618500

[57] Jung AM Dennis LK Jacobs ET Wondrak GT. Sun sensitivity and sun protective behaviors during sun exposure among indoor office workers in the American Midwest. Photodermatol Photoimmunol Photomed. (2018) 34:393–9. doi: 10.1111/phpp.12403, PMID: 29888418

[58] Modenese A Korpinen L Gobba F. Solar radiation exposure and outdoor work: an underestimated occupational risk. Int J Environ Res Public Health. (2018) 15:2063. doi: 10.3390/ijerph15102063, PMID: 30241306 PMC6209927

[59] Zink A Ring J. Images in clinical medicine. Phototoxic dermatitis. N Engl J Med. (2014) 371:559. doi: 10.1056/NEJMicm1315566 25099580

[60] Hajdarevic S Hvidberg L Lin Y Donnelly C Gavin A Lagerlund M. Awareness of sunburn in childhood, use of sunbeds and change of moles in Denmark, Northern Ireland, Norway and Sweden. Eur J Pub Health. (2016) 26:29–35. doi: 10.1093/eurpub/ckv112, PMID: 26082445

[61] Kearney GD Xu X Balanay JA Becker AJ. Sun safety among farmers and farmworkers: a review. J Agromedicine. (2014) 19:53–65. doi: 10.1080/1059924x.2013.855691 24417532

[62] Rocholl M Ludewig M John SM Bitzer EM Wilke A. Outdoor workers' perceptions of skin cancer risk and attitudes to sun-protective measures: a qualitative study. J Occup Health. (2020) 62:e12083. doi: 10.1002/1348-9585.12083, PMID: 31478315 PMC6970388

[63] Smit-Kroner C Brumby S. Farmers sun exposure, skin protection and public health campaigns: an Australian perspective. Prev Med Rep. (2015) 2:602–7. doi: 10.1016/j.pmedr.2015.07.004, PMID: 26844126 PMC4721376

[64] Reiter RJ. Melatonin: the chemical expression of darkness. Mol Cell Endocrinol. (1991) 79:C153–8. doi: 10.1016/0303-7207(91)90087-9, PMID: 1936532

[65] Reiter RJ Tan DX Galano A. Melatonin: exceeding expectations. Physiology (Bethesda). (2014) 29:325–33. doi: 10.1152/physiol.00011.2014, PMID: 25180262

[66] Adamsson M Laike T Morita T. Annual variation in daily light exposure and circadian change of melatonin and cortisol concentrations at a northern latitude with large seasonal differences in photoperiod length. J Physiol Anthropol. (2016) 36:6. doi: 10.1186/s40101-016-0103-9, PMID: 27435153 PMC4952149

[67] Cole RJ Kripke DF Wisbey J Mason WJ Gruen W Hauri PJ. Seasonal variation in human illumination exposure at two different latitudes. J Biol Rhythm. (1995) 10:324–34. doi: 10.1177/074873049501000406 8639941

[68] Cutolo M Maestroni GJ Otsa K Aakre O Villaggio B Capellino S. Circadian melatonin and cortisol levels in rheumatoid arthritis patients in winter time: a north and South Europe comparison. Ann Rheum Dis. (2005) 64:212–6. doi: 10.1136/ard.2004.023416, PMID: 15647428 PMC1755372

[69] Ghareghani M Zibara K Rivest S. Melatonin and vitamin D, two sides of the same coin, better to land on its edge to improve multiple sclerosis. Proc Natl Acad Sci U S A. (2023) 120:e2219334120. doi: 10.1073/pnas.2219334120, PMID: 36972442 PMC10083587

[70] Ghareghani M Reiter RJ Zibara K Farhadi N. Latitude, vitamin D, melatonin, and gut microbiota act in concert to initiate multiple sclerosis: a new mechanistic pathway. Front Immunol. (2018) 9:2484. doi: 10.3389/fimmu.2018.02484, PMID: 30459766 PMC6232868

[71] Simonneaux V Ribelayga C. Generation of the melatonin endocrine message in mammals: a review of the complex regulation of melatonin synthesis by norepinephrine, peptides, and other pineal transmitters. Pharmacol Rev. (2003) 55:325–95. doi: 10.1124/pr.55.2.2, PMID: 12773631

[72] Brainard GC Hanifin JP Greeson JM Byrne B Glickman G Gerner E. Action spectrum for melatonin regulation in humans: evidence for a novel circadian photoreceptor. J Neurosci. (2001) 21:6405–12. doi: 10.1523/jneurosci.21-16-06405.2001, PMID: 11487664 PMC6763155

[73] McDougal DH Gamlin PD. The influence of intrinsically-photosensitive retinal ganglion cells on the spectral sensitivity and response dynamics of the human pupillary light reflex. Vis Res. (2010) 50:72–87. doi: 10.1016/j.visres.2009.10.012, PMID: 19850061 PMC2795133

[74] Aoki H Yamada N Ozeki Y Yamane H Kato N. Minimum light intensity required to suppress nocturnal melatonin concentration in human saliva. Neurosci Lett. (1998) 252:91–4. doi: 10.1016/s0304-3940(98)00548-5, PMID: 9756329

[75] Mack JM Schamne MG Sampaio TB Pértile RA Fernandes PA Markus RP. Melatoninergic system in Parkinson's disease: from neuroprotection to the management of motor and nonmotor symptoms. Oxidative Med Cell Longev. (2016) 2016:1–31. doi: 10.1155/2016/3472032 PMC5088323 27829983

[76] Pazarci P Kaplan H Alptekin D Yilmaz M Lüleyap U Singirik E. The effects of daylight exposure on melatonin levels, kiss 1 expression, and melanoma formation in mice. Croat Med J. (2020) 61:55–61. doi: 10.3325/cmj.2020.61.55, PMID: 32118379 PMC7063558

[77] Poza JJ Pujol M Ortega-Albás JJ Romero O. Melatonin in sleep disorders. Neurologia (Engl Ed). (2022) 37:575–85. doi: 10.1016/j.nrleng.2018.08.004 36064286

[78] Walton JC Weil ZM Nelson RJ. Influence of photoperiod on hormones, behavior, and immune function. Front Neuroendocrinol. (2011) 32:303–19. doi: 10.1016/j.yfrne.2010.12.003, PMID: 21156187 PMC3139743

[79] Brooks PL Peever JH. Glycinergic and GABA(a)-mediated inhibition of somatic motoneurons does not mediate rapid eye movement sleep motor atonia. J Neurosci. (2008) 28:3535–45. doi: 10.1523/jneurosci.5023-07.2008, PMID: 18385312 PMC6671096

[80] Brooks PL Peever JH. Impaired GABA and glycine transmission triggers cardinal features of rapid eye movement sleep behavior disorder in mice. J Neurosci. (2011) 31:7111–21. doi: 10.1523/jneurosci.0347-11.2011, PMID: 21562273 PMC6703223

[81] Brooks PL Peever JH. Identification of the transmitter and receptor mechanisms responsible for REM sleep paralysis. J Neurosci. (2012) 32:9785–95. doi: 10.1523/jneurosci.0482-12.2012, PMID: 22815493 PMC6621291

[82] Niles L. Melatonin interaction with the benzodiazepine-GABA receptor complex in the CNS. Adv Exp Med Biol. (1991) 294:267–77. doi: 10.1007/978-1-4684-5952-4_24, PMID: 1722943

[83] Peever J Luppi PH Montplaisir J. Breakdown in REM sleep circuitry underlies REM sleep behavior disorder. Trends Neurosci. (2014) 37:279–88. doi: 10.1016/j.tins.2014.02.009, PMID: 24673896

[84] Ikram M Park HY Ali T Kim MO. Melatonin as a potential regulator of oxidative stress, and neuroinflammation: mechanisms and implications for the Management of Brain Injury-Induced Neurodegeneration. J Inflamm Res. (2021) 14:6251–64. doi: 10.2147/jir.S334423, PMID: 34866924 PMC8637421

[85] Raha S Robinson BH. Mitochondria, oxygen free radicals, disease and ageing. Trends Biochem Sci. (2000) 25:502–8. doi: 10.1016/s0968-0004(00)01674-1 11050436

[86] Hardeland R. Antioxidative protection by melatonin: multiplicity of mechanisms from radical detoxification to radical avoidance. Endocrine. (2005) 27:119–30. doi: 10.1385/endo:27:2:119, PMID: 16217125

[87] Hardeland R Pandi-Perumal SR. Melatonin, a potent agent in antioxidative defense: actions as a natural food constituent, gastrointestinal factor, drug and prodrug. Nutr Metab (Lond). (2005) 2:22. doi: 10.1186/1743-7075-2-22, PMID: 16153306 PMC1262766

[88] Hardeland R. Melatonin: signaling mechanisms of a pleiotropic agent. Biofactors. (2009) 35:183–92. doi: 10.1002/biof.23, PMID: 19449447

[89] Reiter RJ Rosales-Corral S Tan DX Jou MJ Galano A Xu B. Melatonin as a mitochondria-targeted antioxidant: one of evolution's best ideas. Cell Mol Life Sci. (2017) 74:3863–81. doi: 10.1007/s00018-017-2609-7, PMID: 28864909 PMC11107735

[90] Bazzani V Equisoain Redin M McHale J Perrone L Vascotto C. Mitochondrial DNA repair in neurodegenerative diseases and ageing. Int J Mol Sci. (2022) 23:11391. doi: 10.3390/ijms231911391, PMID: 36232693 PMC9569545

[91] Zhang J Shi Y. In search of the holy grail: toward a unified hypothesis on mitochondrial dysfunction in age-related diseases. Cell. (2022) 11:1906. doi: 10.3390/cells11121906, PMID: 35741033 PMC9221202

[92] Li Y Yang Y Zhao A Luo N Niu M Kang W. Parkinson's disease peripheral immune biomarker profile: a multicentre, cross-sectional and longitudinal study. J Neuroinflammation. (2022) 19:116. doi: 10.1186/s12974-022-02481-3, PMID: 35610646 PMC9131564

[93] Stokholm MG Iranzo A Østergaard K Serradell M Otto M Svendsen KB. Assessment of neuroinflammation in patients with idiopathic rapid-eye-movement sleep behaviour disorder: a case-control study. Lancet Neurol. (2017) 16:789–96. doi: 10.1016/s1474-4422(17)30173-4, PMID: 28684245

[94] Hardeland R. Melatonin and inflammation-story of a double-edged blade. J Pineal Res. (2018) 65:e12525. doi: 10.1111/jpi.12525, PMID: 30242884

[95] Lim HD Kim YS Ko SH Yoon IJ Cho SG Chun YH. Cytoprotective and anti-inflammatory effects of melatonin in hydrogen peroxide-stimulated CHON-001 human chondrocyte cell line and rabbit model of osteoarthritis via the SIRT1 pathway. J Pineal Res. (2012) 53:225–37. doi: 10.1111/j.1600-079X.2012.00991.x, PMID: 22507555

[96] Rusanova I Martínez-Ruiz L Florido J Rodríguez-Santana C Guerra-Librero A Acuña-Castroviejo D. Protective effects of melatonin on the skin: future perspectives. Int J Mol Sci. (2019) 20:4948. doi: 10.3390/ijms20194948, PMID: 31597233 PMC6802208

[97] Yildirim S Ozkan A Aytac G Agar A Tanriover G. Role of melatonin in TLR4-mediated inflammatory pathway in the MTPT-induced mouse model. Neurotoxicology. (2022) 88:168–77. doi: 10.1016/j.neuro.2021.11.011, PMID: 34808223

[98] Anderson KN Shneerson JM. Drug treatment of REM sleep behavior disorder: the use of drug therapies other than clonazepam. J Clin Sleep Med. (2009) 5:235–9. doi: 10.5664/jcsm.27492 19960644 PMC2699168

[99] Boeve BF Silber MH Ferman TJ. Melatonin for treatment of REM sleep behavior disorder in neurologic disorders: results in 14 patients. Sleep Med. (2003) 4:281–4. doi: 10.1016/s1389-9457(03)00072-8 14592300

[100] Kunz D Bes F. Melatonin effects in a patient with severe REM sleep behavior disorder: case report and theoretical considerations. Neuropsychobiology. (1997) 36:211–4. doi: 10.1159/000119383 9396020

[101] Kunz D Bes F. Melatonin as a therapy in REM sleep behavior disorder patients: an open-labeled pilot study on the possible influence of melatonin on REM-sleep regulation. Mov Disord. (1999) 14:507–11. doi: 10.1002/1531-8257(199905)14:3<507::aid-mds1021>3.0.co;2-8, PMID: 10348479

[102] Kunz D Bes F. Twenty years after: another case report of melatonin effects on REM sleep behavior disorder, using serial dopamine transporter imaging. Neuropsychobiology. (2017) 76:100–4. doi: 10.1159/000488893, PMID: 29860260

[103] Kunz D Mahlberg R. A two-part, double-blind, placebo-controlled trial of exogenous melatonin in REM sleep behaviour disorder. J Sleep Res. (2010) 19:591–6. doi: 10.1111/j.1365-2869.2010.00848.x, PMID: 20561180

[104] Kunz D Stotz S Bes F. Treatment of isolated REM sleep behavior disorder using melatonin as a chronobiotic. J Pineal Res. (2021) 71:e12759. doi: 10.1111/jpi.12759, PMID: 34309908

[105] McCarter SJ Boswell CL St Louis EK Dueffert LG Slocumb N Boeve BF. Treatment outcomes in REM sleep behavior disorder. Sleep Med. (2013) 14:237–42. doi: 10.1016/j.sleep.2012.09.018, PMID: 23352028 PMC3617579

[106] Schaefer C Kunz D Bes F. Melatonin effects in REM sleep behavior disorder associated with obstructive sleep Apnea syndrome: a case series. Curr Alzheimer Res. (2017) 14:1084–9. doi: 10.2174/1567205014666170523094938, PMID: 28545360

[107] Takeuchi N Uchimura N Hashizume Y Mukai M Etoh Y Yamamoto K. Melatonin therapy for REM sleep behavior disorder. Psychiatry Clin Neurosci. (2001) 55:267–9. doi: 10.1046/j.1440-1819.2001.00854.x 11422870

[108] Brunani A Perna S Soranna D Rondanelli M Zambon A Bertoli S. Body composition assessment using bioelectrical impedance analysis (BIA) in a wide cohort of patients affected with mild to severe obesity. Clin Nutr. (2021) 40:3973–81. doi: 10.1016/j.clnu.2021.04.033, PMID: 34139470

[109] Misra D Fielding RA Felson DT Niu J Brown C Nevitt M. Risk of knee osteoarthritis with obesity, sarcopenic obesity, and sarcopenia. Arthritis Rheumatol. (2019) 71:232–7. doi: 10.1002/art.40692, PMID: 30106249 PMC6374038

[110] Takesian M Santo MA Gadducci AV Santarém GCF Greve J Silva PR. Trunk body mass index: a new reference for the assessment of body mass distribution. Arq Bras Cir Dig. (2018) 31:1362. doi: 10.1590/0102-672020180001e1362, PMID: 29947696 PMC6050002

[111] Baig F Lawton MA Rolinski M Ruffmann C Klein JC Nithi K. Personality and addictive behaviours in early Parkinson's disease and REM sleep behaviour disorder. Parkinsonism Relat Disord. (2017) 37:72–8. doi: 10.1016/j.parkreldis.2017.01.017, PMID: 28173973 PMC5380654

[112] Haba-Rubio J Frauscher B Marques-Vidal P Toriel J Tobback N Andries D. Prevalence and determinants of rapid eye movement sleep behavior disorder in the general population. Sleep. (2018) 41:zsx197. doi: 10.1093/sleep/zsx197, PMID: 29216391

[113] Long K Wan C Xiang Y Liu J Xu Q Sun Q. Study on the clinical features of Parkinson's disease with probable rapid eye movement sleep behavior disorder. Front Neurol. (2020) 11:979. doi: 10.3389/fneur.2020.00979, PMID: 33041969 PMC7517295

[114] Jun JS Kim R Jung HM Byun JI Seok JM Kim TJ. Emotion dysregulation in idiopathic rapid eye movement sleep behavior disorder. Sleep. (2020) 43:zsz224. doi: 10.1093/sleep/zsz224, PMID: 31553439

[115] Kataoka H Sugie K. Risk for later rapid eye movement sleep behavior disorder in Parkinson's disease: a 6-year prospective study. Int J Neurosci. (2020) 130:237–42. doi: 10.1080/00207454.2019.1667796, PMID: 31516060

[116] McDade EM Boot BP Christianson TJ Pankratz VS Boeve BF Ferman TJ. Subtle gait changes in patients with REM sleep behavior disorder. Mov Disord. (2013) 28:1847–53. doi: 10.1002/mds.25653, PMID: 24130124 PMC3952497

[117] Baglioni C Nanovska S Regen W Spiegelhalder K Feige B Nissen C. Sleep and mental disorders: a meta-analysis of polysomnographic research. Psychol Bull. (2016) 142:969–90. doi: 10.1037/bul0000053, PMID: 27416139 PMC5110386

[118] Nutt D Wilson S Paterson L. Sleep disorders as core symptoms of depression. Dialogues Clin Neurosci. (2008) 10:329–36. doi: 10.31887/DCNS.2008.10.3/dnutt, PMID: 18979946 PMC3181883

[119] Horváth A Montana X Lanquart JP Hubain P Szűcs A Linkowski P. Effects of state and trait anxiety on sleep structure: a polysomnographic study in 1083 subjects. Psychiatry Res. (2016) 244:279–83. doi: 10.1016/j.psychres.2016.03.001, PMID: 27512915

[120] Saunamäki T Jehkonen M. Depression and anxiety in obstructive sleep apnea syndrome: a review. Acta Neurol Scand. (2007) 116:277–88. doi: 10.1111/j.1600-0404.2007.00901.x 17854419

