# Supplementary figures and images for: Causal associations between modifiable risk factors and isolated REM sleep behavior disorder: a mendelian randomization study
Source: Front Neurol. 2024 Feb 7;15:1321216. doi: 10.3389/fneur.2024.1321216 (PMC10880103; doi:10.3389/fneur.2024.1321216)

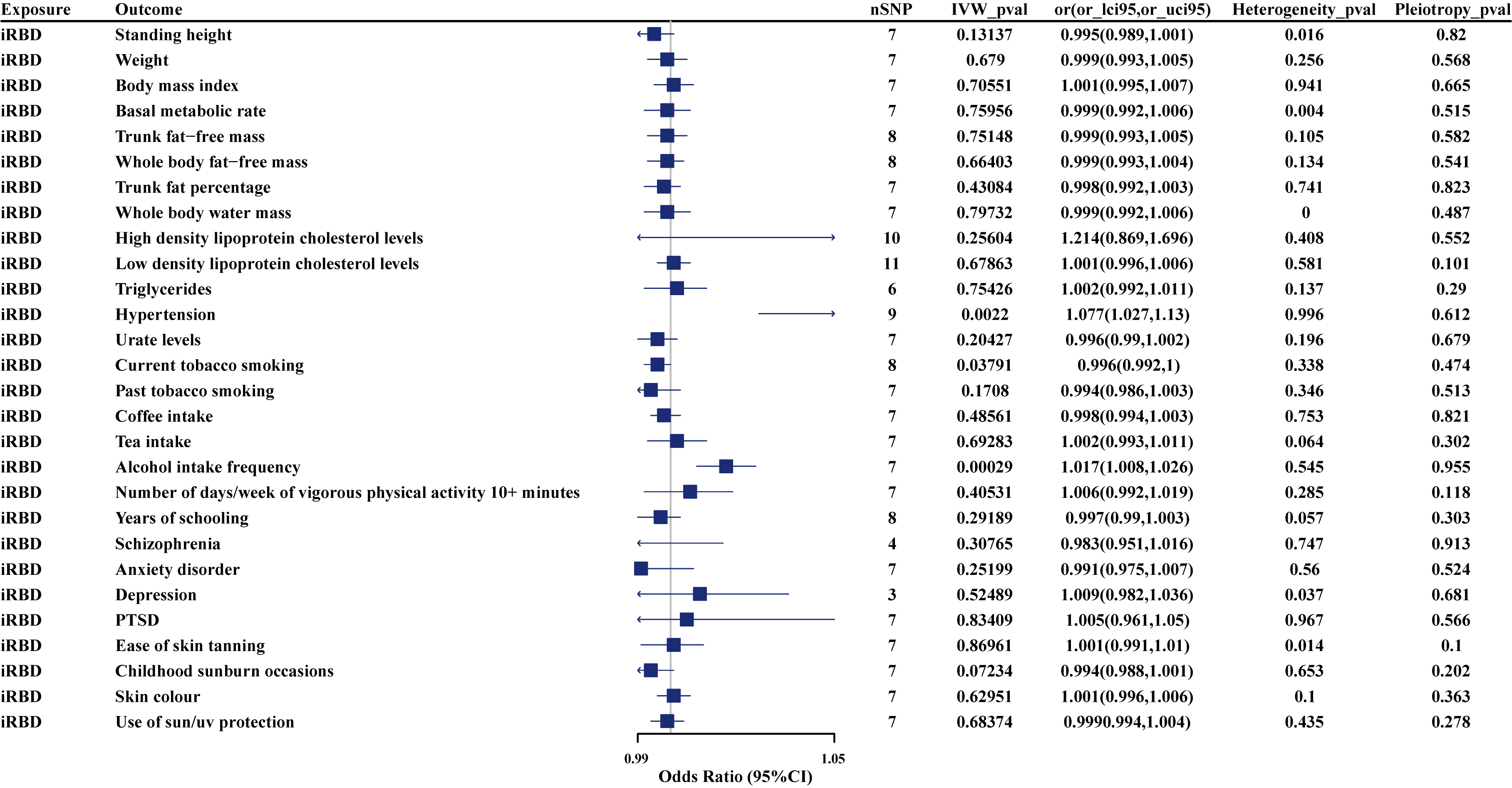

Supplement: Supplementary file 1 [file Image_1.TIF]
